# Modeling and Calibration of a Novel One-Mirror Galvanometric Laser Scanner

**DOI:** 10.3390/s17010164

**Published:** 2017-01-15

**Authors:** Chengyi Yu, Xiaobo Chen, Juntong Xi

**Affiliations:** 1 State Key Laboratory of Mechanical System and Vibration, Shanghai Jiaotong University, Shanghai 200240, China; jg5bvictor@sjtu.edu.cn (C.Y.); xiaoboc@sjtu.edu.cn (X.C.); 2 Shanghai Key Laboratory of Advanced Manufacturing Environment, Shanghai 200030, China

**Keywords:** galvanometric laser scanner, calibration, the screw theory, laser stripe sensor

## Abstract

A laser stripe sensor has limited application when a point cloud of geometric samples on the surface of the object needs to be collected, so a galvanometric laser scanner is designed by using a one-mirror galvanometer element as its mechanical device to drive the laser stripe to sweep along the object. A novel mathematical model is derived for the proposed galvanometer laser scanner without any position assumptions and then a model-driven calibration procedure is proposed. Compared with available model-driven approaches, the influence of machining and assembly errors is considered in the proposed model. Meanwhile, a plane-constraint-based approach is proposed to extract a large number of calibration points effectively and accurately to calibrate the galvanometric laser scanner. Repeatability and accuracy of the galvanometric laser scanner are evaluated on the automobile production line to verify the efficiency and accuracy of the proposed calibration method. Experimental results show that the proposed calibration approach yields similar measurement performance compared with a look-up table calibration method.

## 1. Introduction

In recent decades, the laser stripe sensor has been widely used and is becoming promising in many industrial applications, such as a seam tracking system [1], a weld quality inspecting system [2] and an inspection system of automobile assembly [3], because of its fast acquisition speed, very simple mechanical structure, low cost, and robustness. The main working principle of the laser stripe sensor is that a laser line projector projects a single laser stripe plane onto the object’s surface, forming a light stripe. The light stripe is modulated by the depth of the object’s surface and a camera records the 2D distorted image. The 3D characteristic information of the object’s surface can be derived from the 2D distorted image after performing 3D reconstruction. However, a laser stripe sensor has limited application when a point cloud of geometric samples on the surface of the object is needed to be collected [4]. Taking an inspection system of automobile assembly, for example, the alignments of holes and parts are important, because imprecise positioning of screw holes on a body frame can twist the frame, loosen screws, produce vibration, and deteriorate other qualities. Consequently, the light stripe needs to sweep through the surface of the hole by using mechanical devices to extract its center. The system with such a scanning capability is called a laser scanner.

Tremendous efforts have been devoted to the field of developing and calibrating many laser scanners with different mechanical devices and a wide range of methods have been developed. Li [5,6,7] designed a laser scanner via mounting the laser stripe sensor to the end-effect of a robot. The accuracy of the laser scanner is limited owing to the absolute accuracy of the robot. The laser stripe sensor can also be fixed at the end of the coordinate measuring machine to form a highly accurate laser scanner [8]; however, the coordinate measuring machine is not suitable for being installed on the production line. There is also another form of laser scanner where the position of the projector with respect to the camera coordinate system can be determined via additional reference frame [9,10] or physical constraint [11,12]. Ren [13,14] proposed a linear laser scanner via mounting the laser stripe sensor to a linear rail and the linear rail takes the vision sensor to perform linear scanning. The shortcoming of the linear laser scanner is that its relatively large size and heavy load of the linear rail will limit its industrial applications. Li [15] and Xiao [16] presented a rotational laser scanner by mounting the laser stripe sensor to a turntable. This has the same drawbacks as the linear laser scanner.

The laser scanner using the galvanometric element as its mechanical devices can solve the aforementioned drawbacks. The galvanometric element mainly consists of a precision motor and a reflective mirror. The precision motor guarantees the accuracy of the galvanometric laser scanner. Additionally, the load of the precision motor is just the lightweight mirror and it is much lighter than the aforementioned laser scanners [13,14,15,16]. Furthermore, the galvanometric element is mounted inside the laser scanner, so the size of the galvanometric laser scanner is relatively smaller. However, existing methods [17,18,19,20,21] are only deal with the vision sensor calibration problem with a fixed light stripe plane. Additionally, calibration of a linear laser scanner [13,14] or a rotational laser scanner [15,16] is to determine the movement of the vision sensor, and movement of the whole vision sensor is easily estimated via measuring a static sphere [13,14] or a planar calibration target [15] using the vision sensor. All of the aforementioned calibration methods cannot be used to calibrate the galvanometric laser scanner. Manakov [22] designed a two-mirror galvanometric laser scanner with a point-mark laser projector and proposed a model-driven calibration method. However, the rotational axis that lays on the reflective mirror is assumed in the mathematical model and initial estimations of the intrinsic and extrinsic parameters of the scanning system are calibrated separately. Wagner [23] proposed a look-up table (LUT) to calibrate the two-mirror galvanometric laser scanner and only the pre-defined set of laser rays in LUT can be used in the measuring process. Wissel [24] presented a data-driven learning method for calibrating the two-mirror galvanometric laser scanner, however, a large amount of data is needed to train the data-driven model. The point-mark laser projector along with the two-mirror galvanometric element spends a lot of time to sweeping through the surface of the object, and consequently, the two-mirror galvanometric laser scanner is not suitable for measuring the shape of the object on the production line. Recently, Chi [25] proposed a laser line auto-scanning system for underwater 3D reconstruction, and it overcomes the time-consuming drawback of the point-mark laser scanner [22]. However, the mathematical model [25] assumes that the galvanometer rotation axis should completely coincide with the line intersected by the laser plane and the mirror of the galvanometer. The assumption is relatively harsh for machining and assembly accuracy, because the direction of the laser stripe plane is very hard to measure, and consequently, it is impossible to adjust the incoming laser stripe plane to hit the galvanometer rotation axis. In summary, available mathematical models [22,25] of the galvanometric laser scanner make some assumptions which require the system to be manufactured and assembled well enough, so that the available mathematical models are limited in actual applications.

In this paper, a novel laser scanner is designed via using a one-mirror galvanometer element as the mechanical device. Then the corresponding mathematical model and calibration procedure for the galvanometric laser scanner are proposed. Compared with the linear laser scanner [13,14] and the rotational laser scanner [15,16], the advantage of the proposed galvanometric laser scanner is that its size is very small and its light load increases the life of the motor, so the proposed laser scanner has widespread application prospects in the industrial field, especially for on-line quality inspection. Compared with the mathematical model and the calibration procedure proposed by Manakov [22] and Chi [25], the proposed mathematical model is derived without any position assumptions and all parameters are estimated by minimizing one objective function. The remaining sections are organized as follows: Section 2 introduces the construction and inspecting principle of the galvanometric laser scanner; in Section 3, the mathematical model of the proposed laser scanner is established. Based on the proposed mathematical model, a calibration procedure for the laser scanner is presented. To validate the efficiency and accuracy of the proposed method, contrast experiments are conducted in Section 4; and the paper ends with concluding remarks in Section 5.

## 2. System Working Principles

**Figure 1:**
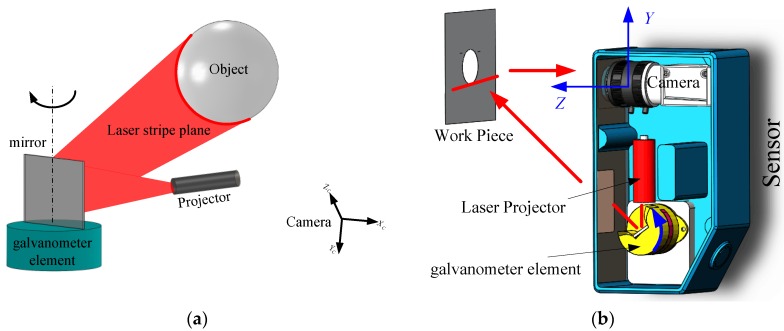
(**a**) Schematic diagram of the one-mirror galvanometric laser scanner; and (**b**) construction of the one-mirror galvanometric laser scanner.

The galvanometric laser scanner is designed using a one-mirror galvanometer element as the mechanical device to collect a point cloud of geometric samples on the surface of the object as shown in Figure 1a. As shown in Figure 1b, the galvanometric laser scanner is mainly comprised of a CCD camera (Basler acA1300/30 µm) with a 16 mm lens, a laser line projector (wavelength of 730 nm, beam width ≤1 mm), and a one-mirror galvanometer element which was made by ourselves using a reflective mirror and a stepper motor. The size of the galvanometric laser scanner is 158 × 116 × 43 mm. Here, there are 23 stripe lines in the view of the sensor, and each pose of the stripe line is fixed in the CCS owing to the high repeatability of the stepper motor. Each rotational step of the galvanometer element is 0.1146°, which is decided by both the speed of the galvanometer element and the frame rate of the camera. 

**Figure 2:**
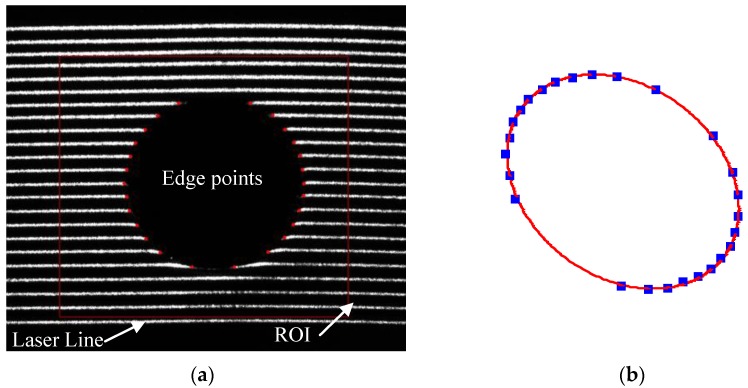
The measuring process of the work piece feature: (**a**) the processed gray image; and (**b**) the corresponding 3D point cloud.

The working principle is as follows: an incoming laser stripe plane, which is projected by a laser line projector, hits the reflective mirror and the light stripe formed by intersecting outgoing laser stripe plane and the object’s surface is captured by the fixed camera. The 3D characteristic information of the object’s surface can be derived from the 2D distorted structured light stripe images after 3D reconstruction. The camera is triggered to acquire pictures continuously when the stepper motor gives a hardware trigger signal. The hardware trigger signal is given when the stepper motor rotates across its zero position. The reflected laser stripe plane scans across the object’s surface along with the rotation of the galvanometer element, and the 3D information of the object’s whole surface can be reconstructed. The measurement result is obtained within one second along with the scanning, so it is very suitable for industrial applications, especially for on-line quality inspection. The measuring process is as shown in Figure 2. In Figure 2a, the edge points of the circle are extracted in the ROI when the laser line scans across the work piece. Figure 2b shows the 3D point cloud of the measured circle after 3D reconstruction. Compared with the Digital TriCam FlexiCam [26], manufactured by Perceptron, Inc., the laser scanner cannot only provide a 3D point cloud without robot motion, but can also be less sensitive to the measurement position and environmental conditions, such as lighting. 

## 3. Modeling and Calibration of the Galvanometric Laser Scanner

The proposed galvanometric laser scanner is calibrated via calibrating the camera first and then estimating the equation of each laser stripe plane with respect to the camera coordinate system (CCS). The camera has been extensively studied in the past decades, and its modeling and calibration techniques are very mature [27,28,29]. The main obstacle is to determine the equation of the reflected laser stripe plane with respect to the CCS at a given rotational angle of the galvanometer element. A mathematic model between the reflected laser plane and the given rotational angle is derived mainly based on the screw theory, and the objective function is established to identify the unknown parameters via minimizing the sum of distances from calibration points (control points) on the laser stripe plane to estimated reflected laser planes. A large number of control points are extracted by employing a plane-constraint-based method.

### 3.1. Camera Calibration

Camera calibration is to estimate intrinsic parameters which reflect the optical characteristic of the camera, and extrinsic parameters that express the pose of the local world coordinate system relative to the camera coordinate system. Zhang [29] presents a flexible camera calibration algorithm in which a planar calibration target is observed by a camera from multiple points of view. Here, the camera is fixed and the 7 × 7 dot array calibration target is freely moved and the relative calibration target poses are unknown. After camera calibration, the pose of each calibration target with respect to the camera coordinate system is determined.

### 3.2. Computing Control Points via the Plane-Constraint-Based Method

Here, a plane-constraint-based approach is proposed to extract a large number of control points on the laser stripe plane to identify the system’s unknown parameters by viewing a planar calibration target from multiple orientations. Compared with the invariance of cross-ratio [19,21] and the invariance of double cross-ratio [20,30], the plane-constraint-based calibration approach extracts the same number of control points as the invariance of double cross-ratio without error propagation.

**Figure 3:**
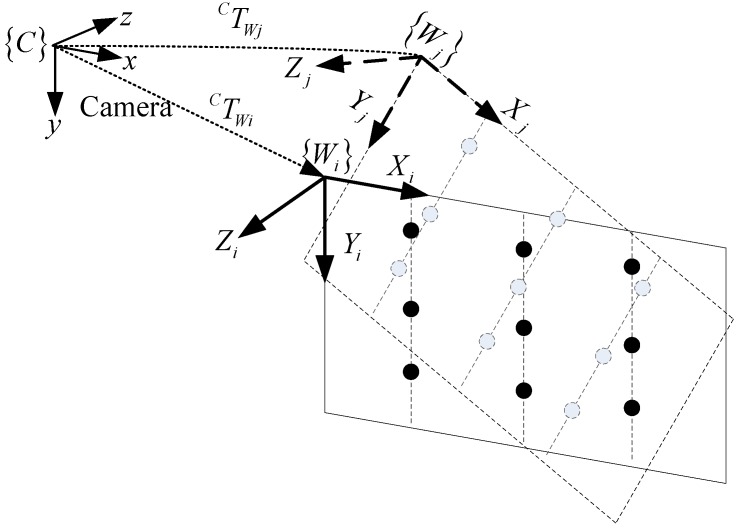
The diagrammatic sketch of transforming the multiple local world coordinate systems into a global camera coordinate system.

Without loss of generality, the local world coordinate system (WCS) of each calibration target is defined as follow: its original point is located at the upper left corner of the planar calibration target, and its X axis is along one side of the target and its Y axis is along the other side, as shown in Figure 3. As a result, the Z coordinates of any points located on the planar target is 0.

(1) Z=0

The camera model can be expanded as:

(2) μP˜i=TCciTwCP˜w=[t11t12t13t14t21t22t23t24t31t32t33t34]P˜w

where TCci and TwC contain intrinsic and extrinsic parameters separately, so they are known after camera calibration. $\tilde{P_{i}}$ is the image plane coordinate of a control point which can be determined by center of mass algorithm [31] and $\tilde{P_{\omega }}$ is the corresponding world coordinates of the control point.

However, TCci·TwC is a 3 × 4 transformation matrix with row rank *r* = 3, so there is no left inverse matrix of TCci·TwC. As a result, the world control point $\tilde{P_{\omega }}$ cannot be derived only from Equation (2). However, all the world control points are constrained on the surface of the planar target, so there is an additional equation (*Z* = 0) for all the world control points. Equations (1) and (2) are combined to form the plane-constraint-based method for extracting control points in the local WCS:

(3) $$\mu \left[\begin{array}{c} {\tilde{P}}_{i}\\ 0 \end{array}\right]=T{\tilde{P}}_{w}=\left[\begin{array}{cccc} t_{11} & t_{12} & t_{13} & t_{14}\\ t_{21} & t_{22} & t_{23} & t_{24}\\ t_{31} & t_{32} & t_{33} & t_{34}\\ 0 & 0 & 1 & 0 \end{array}\right]{\tilde{P}}_{w}$$

where the transformation matrix *T* is a 4 × 4 invertible matrix, and the fourth row of the matrix *T* indicates that all of the world control points are constrained on the *OXY* plane of the local world coordinate system.

The local world control points can be transformed into the global camera coordinate system via extrinsic parameters as shown in Figure 3. The planar calibration target can be placed in multiple poses, so the plane-constraint-based method easily extracts a large number of control points. It is should be noticed that the invariance of the cross-ratio method only extracts, at most, seven control points at each position of the 7 × 7 dot array calibration target, so it is inconvenient for a large number of control points to fit the laser stripe plane. Though the invariance of the double cross-ratio method theoretically extracts all control points on the calibration target, the accuracy of the control points is limited owing to error propagation using the invariance of the double cross-ratio twice.

### 3.3. Mathematical Model of the Galvanometric Laser Scanner

The laser line projector can be modeled as a plane equation and the reflective mirror also can be modeled as a plane equation. The rotational axis of the galvanometric is represented by a spatial line. What is more, all of the equations are expressed in the CCS.

#### 3.3.1. Mathematical Model of Basic Elements

The plane ∏*_p_* equation of the laser line projector (incoming laser plane) is given below

(4) $$a_{p}x_{c}+b_{p}y_{c}+c_{p}z_{c}+d_{p}=0$$

where $\left(a_{p},\;b_{p},\;c_{p},\;d_{p}\right)$ are the estimated plane parameters and $n_{p}=\left(a_{p},\;b_{p},\;c_{p}\right)$ are the normal vector of the plane.

The original plane ∏_0_ equation of the reflective mirror is:

(5) $$a_{0}x_{c}+b_{0}y_{c}+c_{0}z_{c}+d_{0}=0$$

The rotational axis *l* of the galvanometer element is expressed using the directional vector ω and a point P on *l*.

It is should be noticed that there are 11 independent parameters to describe two planes and one spatial line, and no position assumptions are made during the mathematical model of the basic elements. Consequently, machining and assembly errors, which affect the position of basic elements, are considered in the mathematical model. Meanwhile, the position of the laser projector is fixed in the CCS, so the plane equation of the laser line projector is fixed, but unknown, and the equation of the reflective mirror changes along with the rotation of the galvanometer element.

#### 3.3.2. Equation of the Rotated Reflective Mirror

Based on screw theory [32], the transformation matrix T of the rotational axis *l* can be easily expressed as follows, and the twist coordinates ξ for the rotational axis *l* is given below:

(6) $$\xi ={\left[\begin{array}{cc} v & \omega \end{array}\right]}^{T}$$

(7) $$v=-\omega \times P=-\hat{\omega }P$$

where ω is the directional vector of *l* and $P=\left(P_{x},P_{y},P_{z}\right)$ is a point on *l*.

The operator, ∧ (wedge), forms a $R^{4\;\times \;4}$ matrix (the twist $\hat{\xi }$ ) out of a given vector ξ in *R^6^*:

(8) $$\hat{\xi }={\left[\begin{array}{c} v\\ \omega \end{array}\right]}^{\Lambda }=\left[\begin{array}{cc} \hat{\omega } & v\\ 0_{1\times 3} & 0 \end{array}\right]$$

Then the transformation matrix *T* can be expressed via the exponential map from the twist $\hat{\xi }$:

(9) $$T=e^{\hat{\xi }\theta }=\left[\begin{array}{cc} e^{\hat{\omega }\theta } & \left(I-e^{\hat{\omega }\theta }\right)\left(\omega \times v\right)\\ 0_{1\times 3} & 0 \end{array}\right]$$

If the rotational angle θ of the galvanometer element is given, the transformation matrix *T* is determined. The equation of the rotated reflective mirror ∏*_i_* at the given $\theta_{i}$ can be easily expressed as follows:

(10) $${\tilde{n}}_{i}=e^{\hat{\xi }\theta_{i}}{\tilde{n}}_{0}$$

(11) $${\tilde{P}}_{i}=e^{\hat{\xi }\theta_{i}}{\tilde{P}}_{0}$$

where $\tilde{n_{0}}={\left[a_{0},\;b_{0},\;c_{0},\;0\right]}^{T}$ is the homogeneous coordinate of the original reflective mirror’s normal vector in the CCS, and $\tilde{P_{0}}={\left[P_{0x},\;P_{0y},\;P_{0z},\;1\right]}^{T}$ is the homogeneous coordinate of the point $P_{0}$ on the original plane ∏_0_.

#### 3.3.3. Equation of the Reflected Laser Plane

If the incoming laser plane ∏*_p_* and the rotated reflective plane ∏*_i_* are determined, the equation of the reflected laser plane can be given below.

**Figure 4:**
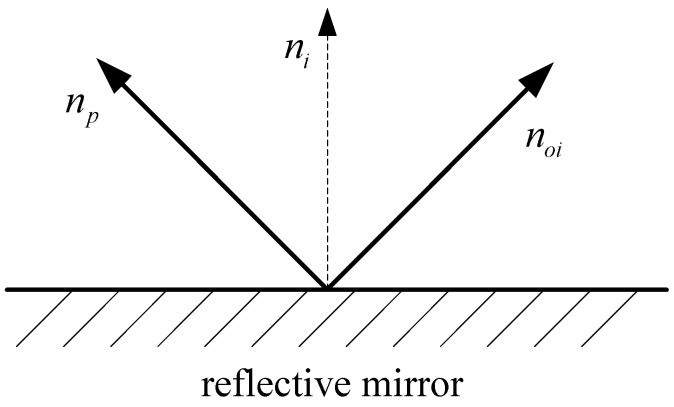
Schematic diagram of determining the reflected laser plane’s normal vector.

The normal vector $n_{oi}$ of the reflected laser plane is estimated as shown in Figure 4 and given below:

(12) $$n_{oi}=2\cdot (n_{i},n_{p})\cdot n_{i}-n_{p}$$

where $n_{p}$ is the normal vector of the incoming laser (laser projector) plane, and $n_{i}$ is the normal vector of the rotated reflective plane ∏*_i_*.

The intersection point of the incoming laser plane and the rotated reflective plane ∏*_i_* can be easily calculated, and then the equation of the reflected laser plane can be easily estimated using the normal vector and a point on the plane. The reflected laser plane at a given $\theta_{i}$ is given below:

(13) $$a_{oi}x_{c}+b_{oi}y_{c}+c_{oi}z_{c}+d_{oi}=0$$

### 3.4. Estimation of the Galvanometric Laser Scanner’s Parameters

There are, in total, 11 independent unknown parameters in the aforementioned model. These parameters can be estimated by minimizing the following objective function:

(14) $$E(X)=\sum_{i=1}^{n}\sum_{j=1}^{m}d_{ij}$$

(15) $$d_{ij}=\left|a_{oi}x_{j}+b_{oi}y_{j}+c_{oi}z_{j}+d_{oi}\right|$$

where $d_{ij}$ is the distance from the control point $P_{j}\left(x_{j},\;y_{j},\;z_{j}\right)$ to the *ith* estimated reflected laser plane, and the point $P_{j}\left(x_{j},\;y_{j},\;z_{j}\right)$ is the point on the *ith* real reflected laser plane. *X* are the 11 independent unknown parameters. A large number of control points are extracted via the plane-constraint-based method as described in Section 3.2.

The Levenberg-Marquardt algorithm [33] is employed to optimize aforementioned parameters.

### 3.5. Summary

**Figure 5:**
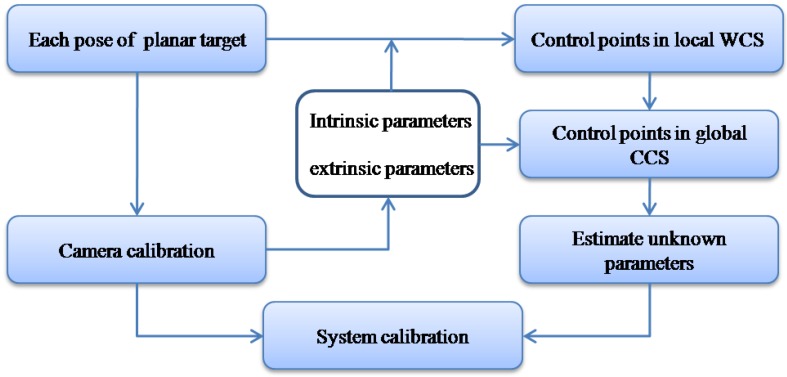
Flowchart of the one-mirror galvanometric laser scanner calibration procedures.

To sum up, the procedure of the proposed calibration method is described in Figure 5 is as follows:

1. The planar calibration target is located at several different poses, and each pose of the planar calibration target is captured by the galvanometric laser scanner. The last two poses are captured twice in two cases, one with no laser stripe line projected onto the planar calibration target for camera calibration and the other with the laser stripe line for projector calibration.
2. The captured images without the laser stripe line are used for camera calibration and both intrinsic parameters and extrinsic parameters are determined.
3. Extracting all image coordinates of control points in two captured images.
4. Correcting distortion for those image coordinates according to the lens distortion model.
5. Calculating control points in the local world coordinate system based on plane-constraint method and transforming the local world coordinate of control points to the global camera coordinate system according the extrinsic parameters.
6. Estimating the 11 independent unknown parameters according to Equation (14).
7. Calibration of the galvanometric laser scanner is completed.

## 4. Experiments

Here, the galvanometric laser scanner is made to be used on the automobile production line. The proposed calibration method is used to calibrate the laser scanner and then repeatability and accuracy of the laser scanner is evaluated via measuring four standard workpieces (a standard sphere feature, a standard hole feature, a standard slot feature, and a standard square feature). The aforementioned features are very common in quality control of body-in-white. Meanwhile, there are 23 stripe lines in the view of the sensor, and each pose of the stripe line is fixed in CCS owing to high repeatability of the stepper motor, so a LUT method can be employed to calibrate the laser scanner. Contrast experiments are conducted to verify the efficiency and accuracy of the proposed calibration method.

### 4.1. System Calibration

The laser scanner is calibrated via minimizing the sum of distances from control points to the estimated reflected laser plane with the Levenberg-Marquardt algorithm. Initial values of unknown parameters can be determined by the structure of the laser scanner. As shown in Figure 1b, the nominal value of the rotational axis *l* can be expressed via the directional vector ω=(1, 0, 0) and a point P=(0,−94,−10). The incoming laser plane ∏*_p_* is adjusted to hit the rotational axis *l*, so the nominal equation of the incoming laser plane is (0, 0, 1, 10). The nominal original plane ∏_0_ equation of the reflective mirror can be determined by the incoming laser plane and the reflected laser plane, so the nominal equation of the plane ∏_0_ is (0, 0.853, 0.477, 87). The aforementioned parameters are good initial guesses for the Levenberg-Marquardt algorithm. The laser scanner is calibrated according to the calibration procedure described in Section 3.5.

### 4.2. Repeatability Evaluation

**Figure 6:**
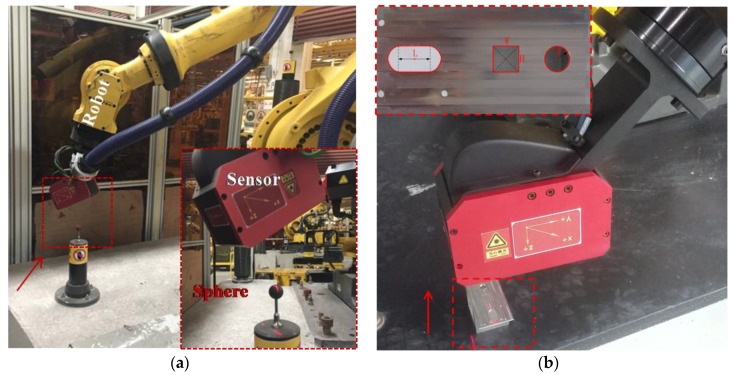
(**a**) The experimental setup in the automobile production line; and (**b**) the experimental setup in the laboratory condition.

**Figure 7:**
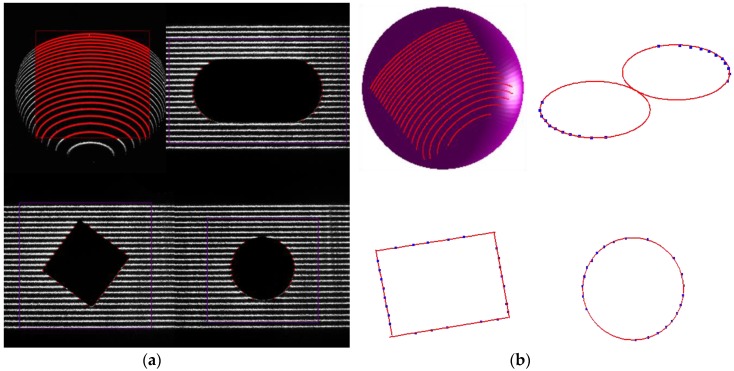
The measuring process of four features: (**a**) the processed gray image; and (**b**) the fitted four features.

**Figure 8:**
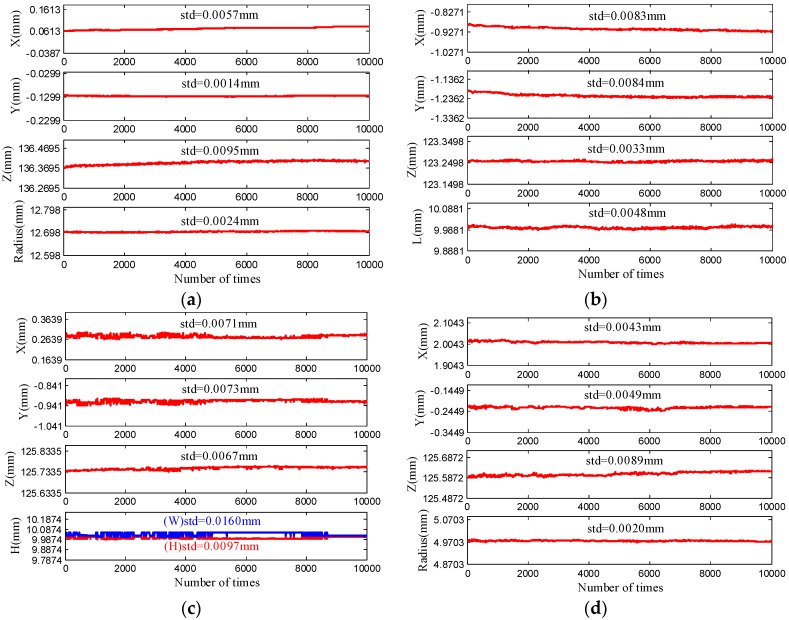
Repeatability evaluation of four features. (**a**) Sphere feature; (**b**) Slot feature; (**c**) Square feature; (**d**) Circle feature.

As shown in Figure 6, the designed galvanometric laser scanner is mounted at the end of the robot to construct a robot-based inspecting system. Coordinates of the spherical centers obtained by the laser scanner are used to calibrate the inspecting system. Here, the standard sphere is a bearing steel ball and is coated with matt material. Other three standard features are manufactured by a five-axis CNC machine (DMG HSC 75 linear, DMG MORI Inc., Bielefeld, Germany), and all standard features are inspected on a CMM (2 + (L/350) μm, Thome Präzision GmbH, Messel, Germany). The definitions of the dimensions of the features are shown in Figure 6b and Table 1 shows true values of the features’ dimensions. In Figure 7a, the surface points of four features are extracted in the ROI and the corresponding 3D points cloud are obtained after calibrating the laser scanner. Figure 7b shows the 3D point cloud and the fitted sphere by a nonlinear least-squares sphere fitting algorithm (TLS_A_) [34], the fitted circle by the geometric circle fitting algorithm [35], and the fitted square by the Hough transform [36]. Repeatability of the laser scanner is evaluated via measuring four features 10,000 times lasting from 10:00 a.m. to 8:00 p.m. with the inspecting system fixed at a certain pose. The influence of the environmental condition changes, such as lighting, on the repeatability of the laser scanner is studied during the long-time repeatability evaluation experiment. The coordinates of four features’ centers with respect to CCS, and the dimension of them, are shown in Figure 8. As indicated in Figure 8, the standard deviation (std) of the laser scanner is less than 0.02 mm and the laser scanner is robust to environmental condition such as lighting. Edges of the square intersect the laser stripe planes at, approximately, a 45° angle, which leads to edge effects in the extraction of the laser stripe center, because the center of mass algorithm is performed for each column of the image independently. Consequently, the laser scanner is less suitable for square features.

### 4.3. Accuracy Evaluation

Here, accuracy of the laser scanner is evaluated via measuring four features from six different poses. The proposed calibration method is compared with other three calibration methods, such as a one-step calibration method (Huynh [19]), a two-step calibration method (Zhou [21]) and other two-step calibration method (plane-constraint-based method). The aforementioned three calibration methods only deal with the vision sensor calibration problem with a fixed light stripe plane. However, there are 23 stripe lines in the view of the sensor, and each pose of the stripe line is fixed in the CCS owing to the high repeatability of the stepper motor. Inspired by LUT, three other calibration methods are repeated 23 times at each pose of the laser stripe plane to complete the calibration of the galvanometric laser scanner and Zhou’s method stands for Zhou’s method with LUT in later paragraph and so on. The LUT method consists of two parts: (1) storing calibration matrices for 23 pre-defined laser planes in a LUT; and (2) usage of the calibration matrix at a pre-defined rotational angle for 3D reconstruction. The LUT method is used as a reference for the proposed model-driven calibration method. Four calibration methods use the same calibration images to complete the calibration procedure and accuracy verification.

**Figure 9:**
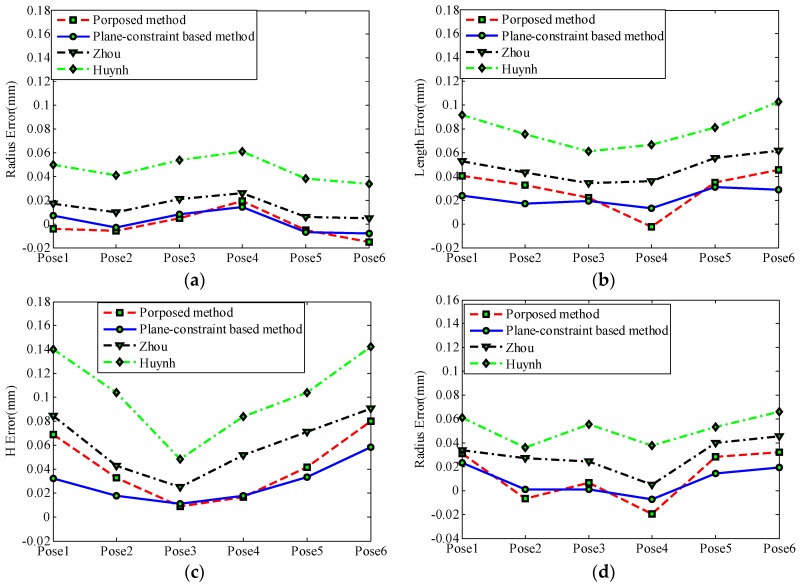
Accuracy comparison among four methods. (**a**) Sphere feature; (**b**) Slot feature; (**c**) Square feature; (**d**) Circle feature.

The root mean square error (RMSE) obtained by estimating the deviation between measurement dimensions and the true value among the proposed calibration method are given in Table 2. As indicated in Table 2 and in Figure 9, the RMSE of the one-step calibration method is much larger than other three two-step calibration methods. This is because the one-step method is based on the homography model [30,37] which neglects the lens distortion when the camera is modeled. The RMSE of the plane-constraint-based calibration method is smaller than that of Zhou’s method, because there are only 14 control points to fit each laser stripe plane in the calibration process described in Section 3.5. As we known, the influence of noise contained in the control points will greatly affect the accuracy of the fitted plane when control points are limited in quantity [20]. The 7 × 7 dot array calibration target should be placed at many positions to extract a large of control points via Zhou’s calibration method, and it is inconvenient to implement. The plane-constraint-based method theoretically extracts all control points on the calibration target by imposing a plane constraint (Z = 0) on them, so it is convenient to estimate the laser stripe plane effectively and accurately. The RMSE of the proposed calibration method (model-driven method) is slightly worse than that of the plane-constraint-based method (LUT method), because the model-driven method estimates the laser stripe planes via the system’s mathematic model, while the LUT method fits each pre-defined laser stripe planes using control points directly. However, the advantages of the proposed method over the LUTs are listed as follows: (1) the LUT method is employed to calibrate only for the pre-defined set of laser stripe planes; (2) the laser scanner needs to be recalibrated only when the structure of the laser scanner changes for the proposed method, while the laser scanner needs to be recalibrated even when the rotational angle changes for the LUT methods.

In summary, repeatability and accuracy of the proposed laser scanner is enough for quality inspecting of the automobile production line. In fact, accuracy of vision sensors is mainly related to the working distance. The proposed laser scanner works at a very close distance (100 mm) with very high accuracy. The rotational laser scanner’s working distance is 110 mm with high accuracy (0.024 mm) [15]. The linear laser scanner’s working distance is 175 mm and the accuracy is about 0.071 mm [14]. There are also laser scanners with long working distance. Chi [25] developed a laser line auto-scanning system for underwater 3D reconstruction at a long working distance (1000 mm) with lower accuracy (0.382 mm). A rotary scanning system is proposed to measure a board from 2000 mm away and the accuracy is about 10 mm [38].

## 5. Conclusions

In this work, a one-mirror galvanometric laser scanner is proposed to collect a point cloud of geometric samples on the surface of the object. Compared with available laser scanners, the proposed one has a relatively small size and its light load increases the life of the motor, so the proposed laser scanner has widespread application prospects in the industrial field. Meanwhile, a mathematical model of the galvanometric laser scanner is derived based mainly on screw theory, and then a calibration procedure is presented. Repeatability and accuracy of the proposed laser scanner are evaluated in the automobile production line and results show that the laser scanner and the calibration procedure are very effective in the industrial applications, especially in the vision inspection system. In addition, the idea of the screw theory-based calibration method can be used for reference when laser scanners with other forms of mechanical devices are calibrated.

## Figures and Tables

**Table 1:** True values of four features’ dimensions.

	Sphere (R)	Slot (L)	Square (H × W)	Circle (R)
True value	12.7080 mm	9.9900 mm	10.01752 mm × 10.03618 mm	4.9862 mm
Std	12.1 μm	0.5 μm	0.8 μm × 2.1 μm	1.5 μm

**Table 2:** Root mean square error (RMSE) comparison among four methods.

Feature Type	Proposed Method (mm)	Plane-Constraint-Based (mm)	Zhou [21] (mm)	Huynh [19] (mm)
Sphere (R)	0.0107	0.0084	0.0162	0.0473
Slot (L)	0.0329	0.0231	0.0483	0.0810
Square (H)	0.0488	0.0323	0.0652	0.1087
Circle (R)	0.0234	0.0139	0.0322	0.0527

## Supplementary Materials

The following are available online at www.mdpi.com/1424-8220/17/1/164/s1, Video S1: The one-mirror galvanometric laser scanner; Video S2: The robot-based inspecting station in the automobile production line.
